# Geographic and Ethnic Variations in Serum Concentrations of Legacy Persistent Organic Pollutants among Men in the Nenets Autonomous Okrug, Arctic Russia

**DOI:** 10.3390/ijerph19031379

**Published:** 2022-01-26

**Authors:** Yulia Varakina, Andrey Aksenov, Dmitry Lakhmanov, Anna Trofimova, Rimma Korobitsyna, Natalia Belova, Dmitry Kotsur, Tatiana Sorokina, Andrej M. Grjibovski, Ludmila Popova, Valery Chashchin, Jon Øyvind Odland, Yngvar Thomassen

**Affiliations:** 1 Arctic Biomonitoring Laboratory, Northern (Arctic) Federal University Named after M. V. Lomonosov, Naberezhnaya Severnoy Dvini 17, 163002 Arkhangelsk, Russia; a.s.aksenov@narfu.ru (A.A.); a.trofimova@narfu.ru (A.T.); r.korobicina@narfu.ru (R.K.); belova-8@mail.ru (N.B.); d.kocur@narfu.ru (D.K.); t.sorokina@narfu.ru (T.S.); andrej.grjibovski@gmail.com (A.M.G.); valerych05@mail.ru (V.C.); yngvar.thomassen@stami.no (Y.T.); 2 Laboratory of Environmental Analytical Chemistry, Core Facility Center “Arktika”, Northern (Arctic) Federal University Named after M. V. Lomonosov, Naberezhnaya Severnoy Dvini 17, 163002 Arkhangelsk, Russia; d.lahmanov@narfu.ru; 3 Northern State Medical University, Troitskiy Ave. 51, 163000 Arkhangelsk, Russia; 4 N. Laverov Federal Center for Integrated Arctic Research, Ural Branch of the Russian Academy of Sciences, Naberezhnaya Severnoy Dvini 23, 163000 Arkhangelsk, Russia; 5 Department of Epidemiology and Modern Vaccination Technology, I.M. Sechenov First Moscow State Medical University (Sechenov University), Trubetskaya Str., 8-2, 119991 Moscow, Russia; 6 West Kazakhstan Marat Ospanov Medical University, Aktobe 0300190, Kazakhstan; 7 Department of Chemistry and Chemical Ecology, Northern (Arctic) Federal University Named after M. V. Lomonosov, Naberezhnaya Severnoy Dvini 17, 163002 Arkhangelsk, Russia; lf.popova@narfu.ru; 8 North-Western State Medical University Named after I. I. Mechnikov, Kirochnaya ul. 41, 191015 Saint-Petersburg, Russia; 9 Institute of Ecology, National Research University Higher School of Economics, Myasnitskaya Str. 20, 101000 Moscow, Russia; 10 Department of Public Health and Nursing, Norwegian University of Science and Technology, 7491 Trondheim, Norway; jon.o.odland@ntnu.no; 11 Department of General Hygiene, I.M. Sechenov First Moscow State Medical University (Sechenov University), Trubetskaya Str., 8-2, 119992 Moscow, Russia; 12 National Institute of Occupational Health, Gydas vei 8, N-0304 Oslo, Norway

**Keywords:** indigenous peoples, biomonitoring, Russian Arctic, men, PCBs, pesticides

## Abstract

The overwhelming majority of Arctic biomonitoring studies in humans include either pregnant or non-pregnant women of reproductive age while little attention is paid to toxic compounds concentrations in men. This study contributes with information of the present amounts of persistent organic pollutants (POPs) in men living in Arctic Russia. We studied the serum concentrations of 11 polychlorinated biphenyl (PCB) congeners and 17 organochlorine pesticides (OCPs) and some of their metabolites in samples collected from 92 adult men (mean age 43 years) from seven different settlements in Nenets Autonomous Okrug (NAO). The median concentrations of individual PCB congeners increased in the order PCB 183, PCB 180, PCB 118, PCB 138, PCB 153. The concentrations of o, p′-DDD, p, p′-DDD, aldrin, mirex and 1,2,3,5-TCB were in most cases below the quantification limit. The observed concentrations of PCBs and chlorinated pesticides were in the same range as those found in similar groups of women of these territories, but lower than of men in other Arctic countries. However, significant geographic differences between the settlements were observed with exceptionally high concentrations of PCBs in the Islands group. The highest serum ∑PCBs and β-HCH levels were observed in adult males aged 60–78 years. We found significant variations in serum concentrations of POPs across settlements and ethnic groups with exceptionally high concentrations of PCBs among the residents of the Arctic islands. At the same time, our findings suggest a considerable decrease in serum concentration of POPs over the last decade.

## 1. Introduction

Persistent organic pollutants (POPs) are hazardous toxic substances with a high affinity for lipids and long half-lives [1]. Some of these compounds, for example HCB or HCH, preferentially condense at temperatures about −30 °C [2].

POPs enter the Arctic ecosystem through atmospheric circulation, oceanic currents, river flow and biological pathways with the risk for negative consequences for human health and the environment [3].

Legacy POPs have been extensively monitored in the Arctic over the last decades, but most of the research have concentrated on the POPs contents in biological fluids of either pregnant or non-pregnant women of reproductive age [4,5,6,7,8,9]. However, studies from Greenland and Canada have presented data of POP levels in serum of men [10,11,12]. High levels of serum POPs such as p, p′-DDE and PCBs 118, 153,138,180 and 183 have been reported in men in [10], although the corresponding levels of serum POPs reported from the areas have been shown to be considerably lower than in, for example, northern Italy or southern Sweden [13,14]. A sufficient amount of evidence has been collected on deleterious effects of POPs on men’s health [2,9,13,15].

Our earlier research has demonstrated low levels of POPs in fish muscles and in the serum of women in the NAO, which is in line with the findings from other Arctic settings. Men living in Arctic Russia have significantly lower life expectancy, higher mortality and poorer general health compared to the national average [16]. Moreover, indigenous men in the Arctic have been reported to have poorer health and more health risks than indigenous women [17], warranting more research on both social and environmental determinants of health of men living in the Arctic.

Long-term exposures to organochlorine pollutants may cause gonadotoxic, embryotoxic and mutagenic effects in males [18]. For example, DDT increases the risk of male infertility [19] and PCBs cause inhibition of the production of sex hormones and deteriorate the quality of semen [20,21]. The most recent data on the concentration of POPs in serum of men was obtained from samples collected more than 10 years ago (2001–2010) in Chukotka in the north-east and in NAO in the north-west of Russia [22]. During that period, serum concentrations of DDT and p, p′-DDE decreased, while no changes have been observed for other POPs. The Arctic Biomonitoring Laboratory was established in 2017 to study effects of POPs in traditional food products on human health in Arctic Russia [23]. Our previous studies have indicated that the level of POPs in fish muscles [24] and in the serum of women [8] in NAO are low and similar to the findings from other Arctic countries.

However, the concentrations of legacy POPs in men in Arctic Russia have not been studied for more than a decade.

The aim of this paper was to present geographic and ethnic variations in serum concentrations of POPs among men permanently living in NAO, European Arctic Russia.

## 2. Materials and Methods

### 2.1. Study Design

**Figure 1:**
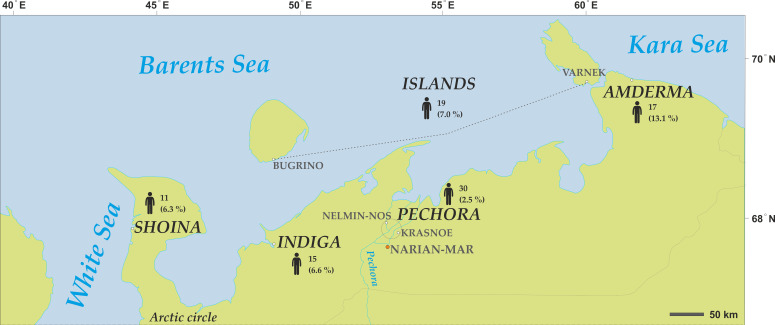
Location of the study settlements, number of study participants and proportion of the total male population. The map was created using CorelDRAW Graphics Suite X4 software (license certificate No 30064931). (https://www.coreldraw.com/) (accessed on 20 July 2021); the topographic base of the map was created with Natural Earth Free Vector and Raster Map Data (https://www.naturalearthdata.com) (accessed on 20 July 2021).

All residents in seven settlements, namely, Bugrino, Varnek, Shoina, Indiga, Amderma, Krasnoe and Nelmin-Nos in NAO were invited to participate in the study. Detailed description of the settlements and recruitment procedures have been presented in our earlier publications [8,25]. Only men were selected for the purpose of this study (n = 92). Participants from the two island locations (Varnek, n = 6 and Kolguev, n = 13) were merged into one group titled the Islands, and participants from the two settlements located on the Pechora river bank (Nelmin-Nos, n = 22 and Krasnoe, n = 8) were merged into one group titled Pechora, considering similarities in environmental, social and dietary characteristics [8,26] (Figure 1).

Ethnic background was registered as reported by the responders. Weight and height were measured, and body mass index (BMI) was calculated.

### 2.2. Serum Samples

A fasting blood sample from the cubital vein was taken from participants for POPs measurements: p, p′-DDE, p, p′-DDD, o, p′-DDE, o, p′-DDD, hexachlorobenzene (HCB), α-hexachlorocyclohexane (α-HCH), β-hexachlorocyclohexane (β-HCH), γ-hexachlorocyclohexane (γ-HCH), heptachlor, cis-chlordane, trans-chlordane, cis-nonachlor, trans-nonachlor, aldrin, mirex, 1,2,3,5-tetrachlorobenzene (1,2,3,5-TCB) and 1,2,4,5-tetrachlorobenzene (1,2,4,5-TCB), as well as the polychlorinated biphenyls (PCBs): 28, 52, 101, 105, 118, 123, 128, 138, 153, 180, 183.

For whole blood sampling we used 9 mL vacutainers Improvacuter (Guangzhou, China), Sarstedt pipettes (Nümbrecht, Germany) for further blood processing and vials (Glasstechnik Grafenroda, Geratal, Germany). A whole blood sample was taken from each examined person, from which a blood serum sample was obtained after centrifugation (3000 rpm). Serum was then transferred to 10 mL vials (Glasstechnik Grafenroda, Geratal, Germany) and frozen to −25 °C before transportation to NaArFU in medical cooler bags for long-term storage at −25 °C until analysis.

### 2.3. Determination of Contaminants and Total Lipids

The analysis of the following POPs set in analytes was carried out in the Core Facility Center “Arktika” of NArFU named after M.V. Lomonosov on a 7890 A gas chromatograph with an Agilent 7000 series triple quadrupole MS/MS system (Santa Clara, CA, USA) operating in the electron ionization mode (70 eV) and with a capillary column HP-5MSUI (30 m × 0.25 mm × 0.25 μm). Quality assurance and control of the determination of analytes in the sample was made by validation of each daily batch including blanks of hexane and matrix. The matrix blank was obtained by mixing equal parts of different serums. A detailed method and conditions for the determination of POPs are described elsewhere [8]. The yields of contaminants ranged from 86 to 120%.

It should be noted that the limit of detection (LOD) and limit of quantification (LOQ) in ng/g lipids is calculated taking into account the total lipid serum content which is different for each sample. The resulting LOD and LOQ ranges, depending on the minimum and maximum lipid levels, as well as the percentage of detection of analytes in the samples are presented in Table 1.

The concentrations of toxicants are presented as the sum of DDT and its metabolites (ΣDDT), and the sum of indicator congeners of PCBs (ΣPCB_5_), expressed in ng/g lipids serum [27,28]. The total lipid content was assessed considering the quantitative content of cholesterol and triglycerides in blood serum using an automatic biochemical analyzer Random Access A-15 (Biosystems, Barcelona, Spain) at the Central Scientific Research Laboratory at the Northern State Medical University in Arkhangelsk.

### 2.4. Statistical Analysis

Distribution of numeric data was assessed using Shapiro–Wilk tests. Given that serum concentrations of all POPs were skewed to the right, the data were logarithmically transformed and presented as geometric means (GM), minimum and maximum values. All values below LOD were assumed to be equal to ½ LOD. Bivariate associations between concentrations of POPs and place of residence were studied using Kruskal–Wallis tests or one way analysis of variance (ANOVA) depending on the distribution. Mann–Whitney tests and Student’s unpaired *t*-tests were applied to assess ethnic differences.

Independent associations between location, ethnicity and serum concentrations of POPs were studied using multivariable linear regression models on logarithmically transformed data with adjustment for age and BMI. Men living along the Pechora River comprised the reference group. Assumptions of linearity, normal distribution of the residuals, and homoscedasticity were tested graphically. They held for o, p-DDE, p, p′-DDE, ∑DDT, PCB118, PCB138, PCB153, PCB180, ∑PCB, HCB and β-HCH, therefore the analysis was limited to these pollutants. Crude (b_c_) and adjusted (b_a_) regression coefficients with 95% confidence intervals (CI) were calculated. All analyses were performed using IBM SPSS software package, version 23.0 (IBM Corp., Armonk, NY, USA).

The study was approved by the local ethics committee at the Northern State Medical University (protocol no. 06/09-17 of 27 September 2017).

## 3. Results

### 3.1. Study Population

The age of the study participants ranged from 18 to 78 years with a mean of 43 years. The majority (55.4%) of the 92 men were Nenets. The rest were Russians (36.9%), Ukrainians (3.3%), Komi (2.2%), Mari (11%) and Udmurts (1.1%). Among them only Nenets were an indigenous group in NAO; therefore, we grouped participants into Nenets and non-Nenets as in our earlier study [17]. BMI of the participants varied from 19.4 to 40.0 kg/m^2^, with an average of 26.7 kg/m^2^. Study participants from different locations did not differ by any of the studied characteristics except the BMI (Table 2).

### 3.2. Serum concentrations of Lipophilic POPs

We detected quantifiable concentrations of 5 PCB congeners (118, 138, 153, 180, 183), 10 chlorinated pesticides (HCB, β-HCH, trans-nonachlor, aldrin, mirex and 1,2,3,5-TCB) and DDT metabolites (p, p′-DDE. p, p′-DDD, o, p′-DDE, o, p′-DDD). Concentrations of α-HCH, γ-HCH, heptachlor, cis-chlordane, trans-chlordane, cis-nonachlor, 1, 2, 4, 5-TCB and PCBs (28, 52, 101, 105, 123,128) were below the limit of quantitative detection (<LOD) (Table 1). GMs, minimum and maximum serum concentrations of POPs are presented in Table 3.

o, p′-DDE, p, p′-DDE, HCB and β–HCH were detected in 82% or 99% of all samples with GM values of 12.6, 68.3, 61.2 and 15.9 ng/g lipids, respectively. Of the PCBs, PCB 153 was present with highest concentration (22.7 ng/g lipids) and detected in 83.7% of the samples. PCB 138 and PCB 180 were detected in 42.4% and 58.7% of the samples with GM values of 16.9 and 5.82 ng/g lipids, respectively. All other compounds were detected above the LOD in between 2 and 34.8% of the samples.

All further measurements were performed only for POPs above the quantitative detection limit in all studied locations and for which all assumptions for multiple linear regression held. These substances were p, p′-DDE, ∑DDT, PCB 118, PCB 138, PCB 153, PCB 180, ∑PCB, HCB and β-HCH.

### 3.3. Geographic Variations in Serum Concentration of POPs

#### 3.3.1. Crude Analysis

Significant variations in the concentrations of p, p′-DDE, PCB 118, PCB 138, PCB 153, PCB 180, PCB, and HCB were observed between the settlements (Table 4). Concentrations of p, p′-DDE, PCB 138, PCB 153, ∑PCB in the Islands, in Shoina and Amderma were significantly higher than in Pechora. The PCB 118 concentration in Amderma and Indiga was greater compared to the reference group. Concentration of PCB 180 in all settlements exceeded the levels in Pechora. HCB concentration in Pechora was greater than in Shoina and Amderma. At the same time, men living along the Pechora River had lower β-HCH levels than men living in Shoina. Detailed data on crude variations in serum concentration of selected POPs is presented in Table 4.

#### 3.3.2. Adjusted Analysis

Adjustment for age, BMI and ethnic background attenuated the differences in p, p′-DD concentrations between Amderma and Pechora, but mainly between the Islands and the reference group. Geographic variations in serum concentrations of PCB 118, PCB 138, 153, PCB 180, ∑PCB and HCB remained significant while no independent associations between location and β-HCH were observed in multivariable analysis (Table 5).

### 3.4. Ethnic Variations in Serum Concentration of POPs

#### 3.4.1. Crude Analysis

We observed ethnic differences in serum concentration of p, p′-DDE, PCB 118 and HCB (Table 4). Nenets men had higher concentration of HCB, but lower levels of p, p′-DDE and PCB 118.

#### 3.4.2. Adjusted Analysis

Only p, p′-DDE and ∑DDT concentrations remained significantly lower in the Nenets compared to non-Nenets. All other associations decreased to non-significant levels.

Age was significantly associated with PCB 138, PCB 153, PCB 180, ∑PCB and β-HCH while BMI was associated with p, p′-DDE, ∑DDT and β-HCH in multivariable analysis (Table 5).

## 4. Discussion

To our knowledge, this is the first study of the distribution of POPs in men across settlements and ethnic groups in Arctic Russia.

Although recent data on POPs from Russia are available in international literature, these studies included only women [7,8,29]. Studies from Greenland and Canada have reported concentrations of POPs by gender [10,11,12] while the data from NAO [3,30] have not been stratified. Therefore, our results can be used as a starting point for monitoring of POPs in men in European Arctic Russia.

The most detected pesticide in the men’s serum in Arctic Russia is p, p′-DDE, which is similar to the results from the United States, Europe, and China [31]. Geometric mean values for ∑PCB_5_ and DDT in men’s serum were 74.1 and 125 ng/g lipids, respectively (Table 3), which is from 6 to 11 times as low as reported in earlier publications from northwestern Russia [32]. Concentrations of ΣPCB_5_ among Inuits in Canada [10,11] were four times as high as in our study. Residents of Greenland had 40 times as high concentrations of PCBs [12] as the residents of NAO. High levels of PCBs are likely to be associated with the more frequent consumption of sea animals [33,34] in Greenland and Arctic Canada than in NAO.

**Figure 2:**
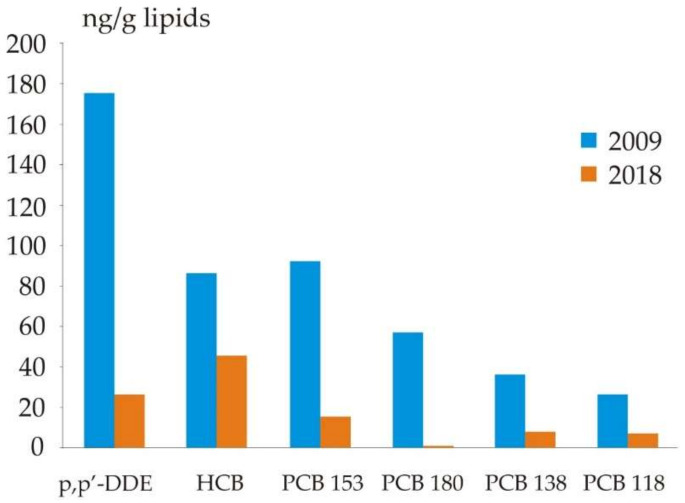
Median serum concentration of individual POPs in men in 2009 [32] and 2018 in Nelmin-Nos, Nenets Autonomous Okrug, Arctic Russia.

The only settlement with available serum concentrations of selected POPs from earlier periods was Nelmin-Nos. Our results from the same age group suggest a decrease in the serum concentrations of p, p′-DDE, HCB, PCBs (153, 180, 138, 118) compared to the results from 2009 ([32], Figure 2).

A decrease in serum concentrations of POPs has also been observed in men in other regions. For example, concentrations of PCB 153 and p, p′-DDE in Greenland decreased annually by 6.67–8.61% and by 6.11–9.52%, respectively, between 1997 and 2007 [33]. In Barcelona, the concentrations of PCB 153, p, p′-DDE, HCB and β-HCH decreased by 10%-75% between 2006 and 2016 [34]. This is most likely a result of adoption of the Stockholm Convention on Persistent Organic Pollutants in 2004 aiming at elimination or limiting the production and use of POPs [9]. Replacement of traditional diet by processed foods including imported food items may also have contributed to the observed trend in POPs concentrations in Arctic Russia.

The main factors associated with the accumulation of POPs are age, BMI, environment and nutrition [14,35,36,37,38,39]. In addition to these main factors, there are ethnic [25] and geographical [33,40] variations. For example, the highest concentrations of p, p′-DDE, PCB 153 were observed in the north of Greenland compared to other parts of the island [40].

Similarly, residents of the Islands and Shoina located in the north and northwest of NAO had higher serum concentrations of p, p′-DDE, which is the most persistent metabolite of DDT [41]. Coastal areas and the islands of NAO were most likely to be contaminated by long-range transport of DDT prior to mitigation measures under the Stockholm Convention. On the other hand, there is a local pollution of the environment with these compounds [38,39], resulting in human body contamination through the local food chains [42,43,44].

High levels of PCBs (PCB 138, PCB 153, PCB 180, ∑PCB) are common in all areas of NAO except Indiga and Pechora. Men living on the islands have higher concentration of PCB 138, PCB 153, PCB 180 than men living in other settlements. This is in line with the findings on serum concentrations of POPs among women from the same settlements [8]. Higher concentrations of PCBs in both men and women living on the islands may be at least partly attributed to consumption of sea mammals [45], which have been shown to have concentrations of PCBs more than 10 times as high as other traditional foods in the Arctic [4,24,40,46]. More research is needed to explain the nutritional factors behind the observed differences in serum concentrations of POPs among the residents of the Arctic islands. Residents of Shoina and Amderma have lower serum levels of than those of Pechora and Indiga. Freshwater fish and dairy products have been previously shown to be associated with HCB concentrations [47,48]. According to the food frequency questionnaire [49], residents of Pechora more frequently consume dairy products, mainly from the dairy factory located nearby.

Ethnic variations in serum concentrations of selected POPs have also been observed in other Arctic settings, potentially reflecting the differences in traditional nutrition. For example, indigenous people in Greenland had higher concentrations of PCB and HCB [12], which was explained by nutritional factors. Indigenous people are more likely to consume traditional foods such as marine fish and mammals that contain higher amounts of POPs than other foods [24,44,50]. In our study, Nenets had lower levels of p, p′-DDE and ∑DDT than non-Nenets while the evidence for other pollutants was inconclusive in multivariable analysis. Thus, we may speculate that Nenets and non-Nenets from the same settlements may have similar food patterns and similar concentrations of POPs as suggested in other studies [26,30].

Age and BMI are other factors that need to be considered. We found positive associations between age, BMI and serum concentrations for half of the studied pollutants. Despite a global decreasing trend in PCBs and OCPs in the environment [51], the highest concentrations are often observed in the elderly due to higher environmental concentrations in the past and long biological half-lives of the POPs. ΣDDT, p, p′-DDE and PCB 138, 153, 180 have the highest level of bioaccumulation in serum [37].

We emphasize that Arctic biomonitoring studies should consider the ethnic and geographic heterogeneity of the population as well as variations in food patterns and potential sources of POPs.

## 5. Conclusions

Serum concentrations of most PCBs and OCPs in the population of NAO are considerably lower than in other parts of the Arctic. Moreover, the levels of POPs in 2018 were substantially lower than in 2009. We observed significant geographic variations in serum concentrations of the PCB congeners 138, 153, 180, p, p′-DDE and HCB while ethnic variations were found only for p, p′-DDE. These differences are likely to be attributed to the differences in nutrition. Our results demonstrate the need for biomonitoring studies for both men and women in Arctic Russia for the evaluation of long-term trends in legacy POPs and new emerging POPs. Further studies should concentrate on the factors behind the observed geographic and ethnic differences.

## Figures and Tables

**Table 1:** Limit of detection (LOD), limit of quantification (LOQ) and the percentage of analytes detected in sample.

Analyte	% > LOD ^a^	LOD		LOQ	
		Range (ng/g Lipid)		Range (ng/g Lipid)	
PCB28	- ^b^	0.40	1.14	1.80	5.07
PCB52	-	2.92	8.24	9.74	27.5
PCB101	-	4.11	11.6	13.7	38.6
PCB105	-	5.92	16.7	19.7	55.6
PCB118	18.5	8.20	19.3	23.9	67.4
PCB123	-	4.43	12.5	14.8	41.6
PCB128	-	4.22	11.9	14.1	39.7
PCB138	42.4	9.50	22.4	27.8	78.4
PCB153	83.7	2.52	5.95	7.38	20.8
PCB180	58.7	1.23	2.90	3.59	10.1
PCB183	5.43	5.49	12.9	16.1	45.3
o, p′-DDE	82.6	2.06	4.86	6.03	17.0
p, p′-DDE	90.2	10.9	25.7	31.9	89.9
o, p′-DDD	6.52	0.84	1.98	2.46	6.94
p, p′-DDD	34.8	4.78	11.3	14.0	39.5
HCB	98.9	3.50	8.25	10.2	28.9
α-HCH	-	5.59	15.8	18.6	52.5
β-HCH	96.7	2.00	4.71	5.84	16.5
γ-HCH	-	3.62	10.2	12.1	34.0
cis-nonachlor	-	3.51	9.91	11.7	33.0
trans-nonachlor	8.70	8.85	20.9	25.9	73.0
cis-chlordane	-	3.51	9.91	11.7	33.0
trans-chlordane	-	8.56	24.1	28.5	80.4
Heptachlor	-	7.68	21.7	25.6	72.2
Aldrin	6.52	9.53	22.5	21.7	61.2
Mirex	2.17	6.10	14.4	17.9	50.3
1,2,3,5-tetrachlorobenzene	22.8	1.09	2.58	3.20	9.02
1,2,4,5-tetrachlorobenzene	-	0.96	2.71	3.20	9.02

^a^ % > LOD—the percentage of analytes detected above the limit of quantitative detection in the sample; ^b^ ”-“—no signal.

**Table 2:** Background characteristics of the study participants, Nenets Autonomous Okrug, Arctic Russia.

Characteristic	Measures	Islands	Shoina	Amderma	Indiga	Pechora	*p*-Value ^a^	Nenets	Non-Nenets	*p*-Value ^b^
	N	19	11	17	15	30		51	41	
Age, years	GM	37.5	44.1	45.0	36.9	40.9	0.502	40.2	41.06	0.801
	Min	18.0	28.0	28.0	19.0	20.0		20.0	18	
	Max	78.0	63.0	72.0	59.0	70.0		78.0	72	
Total lipid, mg/dL	GM	578	599	627	560	595	0.615	588	597	
	Min	395	459	468	414	417		395	414	0.485
	Max	1110	859	977	821	874		1110	977	
BMI, (kg/m^2^)	GM	25.0	27.4	28.3	27.7	25.2	0.018	26.1	26.7	
	Min	19.8	20.5	19.7	19.4	20.8		19.8	19.4	0.719
	Max	34.5	37.1	37.1	36.0	40.0		37.1	40.0	
BMI > 30, n (%)		2 (10.5)	2 (18.2)	8 (47.1)	6 (40)	4 (13.3)		10 (20)	12 (29)	
Proportion of Nenets, n (%)		17 (89.5)	1 (9.10)	5 (29.4)	6 (40.0)	22 (73.3)				

^a^*p*-value for difference between the settlements calculated using one-way ANOVA.; ^b^*p*-value for difference between the settings calculated using Student *t*-test.

**Table 3:** Serum concentrations of POPs (ng/g lipids) in men of the Nenets Autonomous Okrug, Arctic Russia.

Analyte	Measures	Islands	Shoina	Amderma	Indiga	Pechora	*p*-Value ^3^	Total	Nenets	Non-Nenets	*p*-Value ^4^
	N	19	11	17	15	30		92	51	41	
PCBs, ng/g lipid											
PCB 118	GM	8.24	<LOD	14.7	12.3	<LOD		9.08	<LOD	10.5	
	Min	<LOD ^5^	<LOD	<LOD	<LOD	<LOD	<0.001	<LOD	<LOD	<LOD	0.049
	Max	45.0	24.0	42.1	89.0	<LOD		89.0	45.0	89.0	
PCB 138	GM	37.7	30.7	19.1	12.8	<LOD		16.9	16.3	17.7	
	Min	<LOD	<LOD	<LOD	<LOD	<LOD	<0.001	<LOD	<LOD	<LOD	0.489
	Max	515	96.8	60.0	163	54.6		515	515	163	
PCB 153	GM	90.4	41.6	31.5	12.7	8.34		22.7	27.5	17.8	
	Min	<LOD	8.84	<LOD	<LOD	<LOD	<0.001	<LOD	<LOD	<LOD	0.156
	Max	2640	142	83.3	292	77.2		2640	2640	292	
PCB 180	GM	52.9	11.9	15.7	3.32	<LOD		5.82	5.84	5.80	
	Min	6.55	2.22	<LOD	<LOD	<LOD	<0.001	<LOD	<LOD	<LOD	0.102
	Max	1420	37.4	52.4	118	<LOD		1420	1420	118	
PCB 183	GM	6.84	<LOD	<LOD	<LOD	<LOD		<LOD	<LOD	<LOD	
	Min	<LOD	<LOD	<LOD	<LOD	<LOD	-	<LOD	<LOD	<LOD	0.064
	Max	45.6	<LOD	<LOD	<LOD	<LOD		45.6	45.6	6.03	
∑ PCB_5_^1^	GM	222	110	92.2	52.5	33.6		74.1	80.2	67.2	
	Min	28.8	35.1	20.6	18.9	15.0	<0.001	15.0	19.3	15.0	0.165
	Max	4600	257	217	666	137		4600	4600	666	
OCPs ng/g lipid											
o, p-DDE	GM	21.7	23.0	6.57	3.81	18.6		12.6	13.0	12.0	
	Min	2.70	11.8	<LOD	<LOD	<LOD	<0.001	<LOD	<LOD	<LOD	0.407
	Max	61.8	38.5	23.7	24.0	75.2		75.2	65.6	75.2	
p, p′-DDE	GM	121	152	123	41.8	32.5		68.3	48.7	104	
	Min	30.6	43.3	22.7	<LOD	<LOD	<0.001	<LOD	<LOD	16.0	0.009
	Max	467	549	1320	336	278		1320	467	1320	
o, p-DDD	GM	<LOD	<LOD	<LOD	<LOD	0.99		0.92	0.86	1.00	
	Min	<LOD	<LOD	<LOD	<LOD	<LOD	-	<LOD	<LOD	<LOD	0.262
	Max	<LOD	<LOD	<LOD	<LOD	129		129	1.73	129	
p, p′-DDD	GM	<LOD	<LOD	<LOD	<LOD	47.5		9.62	12.1	7.22	
	Min	<LOD	<LOD	<LOD	<LOD	17.3		<LOD	<LOD	<LOD	0.165
	Max	<LOD	<LOD	8.41	6.03	183		183	183	109	
∑ DDT ^2^	GM	161	193	146	55.0	124		125	109	146	
	Min	61.3	72.4	41.1	15.3	36.2	0.002	15.3	15	21.4	0.019
	Max	506	570	1321	342	534		1320	506	1321	
HCB	GM	83.1	26.3	34.2	133	65.1		61.2	81.7	42.8	
	Min	<LOD	10.85	9.4	22.2	12.5	<0.001	<LOD	<LOD	9.38	0.014
	Max	358	109	250	469	713		713	713	370	
β-HCH	GM	13.2	26.4	17.1	19.8	12.7		15.9	13.2	19.9	
	Min	2.75	3.17	4.00	7.21	2.10	0.227	2.10	2.75	2.10	0.006
	Max	31.5	178	118	139	89.2		178	139	178	
Aldrin	GM	18.1	<LOD	<LOD	<LOD	<LOD		<LOD	10.5	<LOD	
	Min	<LOD	<LOD	<LOD	<LOD	<LOD	-	<LOD	<LOD	<LOD	0.138
	Max	987	<LOD	24.6	<LOD	<LOD		987	987	24.6	
Mirex	GM	<LOD	<LOD	<LOD	<LOD	<LOD		<LOD	<LOD	<LOD	
	Min	<LOD	<LOD	<LOD	<LOD	<LOD		<LOD	<LOD	<LOD	0.207
	Max	36.6	<LOD	<LOD	<LOD	<LOD	-	36.6	36.6	7.24	
trans-nonachlor	GM	11.9	9.60	<LOD	<LOD	<LOD		<LOD	9.05	<LOD	
	Min	<LOD	<LOD	<LOD	<LOD	<LOD	-	<LOD	<LOD	<LOD	0.207
	Max	95.7	34.8	<LOD	26.1	<LOD		95.7	95.7	34.8	
1,2,3,5-TCB	GM	<LOD	<LOD	1.73	64.9	<LOD		1.96	1.52	2.70	
	Min	<LOD	<LOD	<LOD	12.5	<LOD	-	<LOD	<LOD	<LOD	0.798
	Max	<LOD	<LOD	10.4	502	<LOD		502	502	250	

^1^ sum PCB# 118, 138, 153, 180, 183; ^2^ sum p, p′-DDE, p, p′-DDD, o. p′-DDE. o, p′-DDD; ^3^ *p*-values calculated by Kruskal–Wallis test; ^4^ *p*-values for difference between the settlements calculated using Mann–Whitney test; ^5^ <LOD—below the limit of quantitative detection [8]; GM—geometric mean.

**Table 4:** Crude coefficients (b_c_) and 95% confidence intervals (CI) for the associations between logarithmically transformed serum concentrations of legacy POPs and selected covariates among men in the Nenets Autonomous Okrug.

Variable	Crude Differences between Pechora and Other Locations								Crude Ethnic Differences		Age		BMI	
	Islands		Shoina		Amderma		Indiga		Nenets vs. non-Nenets					
	b_c_ *	95% CI	b_c_	95% CI	b_c_	95% CI	b_c_	95% CI	b_c_	95% CI	b_c_	95% CI	B *	95% CI
p, p′-DDE	**1.31**	**0.66; 1.96**	**1.55**	**0.76; 2.32**	**1.33**	**0.66; 2.01**	0.25	−0.45; 0.95	**−0.76**	**−1.27; −0.25**	0	−0.02; 0.02	**0.07**	**0.01; 0.13**
∑ DDT	0.26	−0.16; 0.69	0.44	−0.07; 0.95	0.16	−0.28; 0.60	**−0.81**	**−1.27; −0.36**	−0.29	−0.63; 0.04	0.01	0.00; 0.02	0.03	−0.01; 0.06
PCB 118	0.20	−0.12; 0.53	0.13	−0.26; 0.52	**0.78**	**0.44; 1.12**	**0.61**	**0.26; 0.96**	**−0.26**	**−0.51; −0.01**	0.01	0.00; 0.02	0.04	0.01; 0.08
PCB 138	**1.46**	**0.96; 1.96**	**1.26**	**0.66; 1.85**	**0.78**	**0.27; 1.30**	0.38	−0.16; 0.91	−0.08	−0.50; 0.34	**0.02**	**0.01; 0.03**	0.04	−0.01; 0.07
PCB 153	**2.38**	**1.64; 3.13**	**1.61**	**0.71; 2.51**	**1.33**	**0.56; 2.10**	0.42	−0.38; 1.23	0.43	−0.21; 1.08	**0.03**	**0.01; 0.05**	0.01	−0.06; 0.09
PCB 180	**4.14**	**3.50; 4.79**	**2.65**	**1.87; 3.43**	**2.93**	**2.26; 3.60**	**1.37**	**0.68; 2.07**	0.01	−0.80; 0.82	0.02	−0.01; 0.05	0.03	−0.06; 0.12
∑ PCB	**1.89**	**1.39; 2.38**	**1.19**	**0.59; 1.78**	**1.01**	**0.50; 1.52**	0.45	−0.09; 0.98	0.18	−0.28; 0.63	**0.02**	**0.01; 0.03**	0.02	−0.03; 0.08
HCB	0.25	−0.28; 0.77	**−0.91**	**−1.53; −0.28**	**−0.64**	**−1.18; −0.11**	**0.71**	**0.15; 1.27**	**0.65**	**0.24; 1.05**	0.01	−0.01; 0.02	0.02	−0.03; 0.06
β-HCH	0.04	−0.46; 0.54	**0.73**	**0.13; 1.33**	0.30	−0.22; 0.81	0.44	−0.10; 0.98	−0.41	−0.76; −0.05	**0.02**	**0.01; 0.03**	**0.06**	**0.02; 0.10**

* Statistically significant associations are in bold.

**Table 5:** Adjusted coefficients (b_a_) and 95% confidence intervals (CI) for the associations between logarithmically transformed serum concentrations of legacy POPs and selected covariates among men in the Nenets Autonomous Okrug.

Variable	Adjusted Differences between Pechora and Other Locations								Adjusted Ethnic Differences		Age		BMI	
	Islands		Shoina		Amderma		Indiga		Nenets vs. Non-Nenets					
	b_a_ *	95% CI	b_a_ *	95% CI	b_a_ *	95% CI	b_a_ *	95% CI	b_a_ *	95% CI	b_a_ *	95% CI	b_a_ *	95% CI
p, p′-DDE	**1.38**	**0.75;2.02**	**0.91**	**0.08; 1.73**	0.62	−0.10; 1.43	−0.30	−1.01; 0.41	**−0.80**	**−1.32; −0.27**	0.00	−0.02; 0.01	**0.07**	**0.02; 0.13**
∑ DDT	0.33	−0.08; 0.74	0.01	−0.52; 0.54	−0.32	−0.78; 0.15	**−1.13**	**−1.59; −0.68**	**−0.51**	**−0.85; −0.17**	0.01	−0.01; 0.02	**0.04**	**0.01; 0.08**
PCB 118	0.23	−0.11; 0.56	0	−0.44; 0.43	**0.60**	**0.22; 0.98**	**0.50**	**0.12; 0.88**	0.13	−0.41; 0.15	0.01	0.00; 0.02	0.02	−0.01; 0.05
PCB 138	**1.53**	**1.04; 2.01**	**1.13**	**0.50; 1.76**	**0.56**	**0.01; 1.11**	0.33	−0.22; 0.87	−0.07	−0.48; 0.33	**0.02**	**0.01; 0.03**	0.03	−0.01; 0.07
PCB 153	**2.33**	**1.60; 3.06**	**1.82**	**0.89; 2.78**	**1.47**	**0.64; 2.29**	0.70	−0.12; 1.52	0.54	−0.06; 1.15	**0.03**	**0.01; 0.04**	0.00	−0.06; 0.06
PCB 180	**4.24**	**3.58; 4.90**	**2.70**	**1.85; 3.56**	**2.95**	**2.21; 3.70**	**1.48**	**0.74; 2.22**	0.17	−0.38; 0.72	**0.02**	**0.00; 0.04**	0.00	−0.06; 0.05
∑ PCB	**1.90**	**1.42; 2.38**	**1.19**	**0.57; 1.82**	**0.94**	**0.40; 1.49**	0.52	−0.03; 1.01	0.17	−0.23; 0.57	**0.02**	**0.01; 0.03**	0.01	−0.03; 0.05
HCB	0.20	−0.32; 0.73	**−0.73**	**−1.41; −0.05**	**−0.66**	**−1.25; −0.07**	**0.74**	**0.15; 1.33**	0.42	−0.02; 0.86	0.01	0.00; 0.02	0.02	−0.02; 0.07
β-HCH	0.06	−0.40; 0.52	0.39	−0.21; 0.99	−0.15	−0.67; 0.37	0.25	−0.27; 0.76	−0.30	−0.68; 0.09	**0.02**	**0.01; 0.03**	**0.05**	**0.01; 0.09**

* Adjusted for age, body mass index, ethnic background and location. Statistically significant associations are in bold.

## Data Availability

The data presented in this study are available on request from the corresponding author.
